# Discovery of predictors of sudden cardiac arrest in diabetes: rationale and outline of the RESCUED (REcognition of Sudden Cardiac arrest vUlnErability in Diabetes) project

**DOI:** 10.1136/openhrt-2020-001554

**Published:** 2021-02-05

**Authors:** Laura H van Dongen, Peter P Harms, Mark Hoogendoorn, Dominic S Zimmerman, Elisabeth M Lodder, Leen M 't Hart, Ron Herings, Henk C P M van Weert, Giel Nijpels, Karin M A Swart, Amber A van der Heijden, Marieke T Blom, Petra J Elders, Hanno L Tan

**Affiliations:** 1 Clinical and Experimental Cardiology, Amsterdam UMC - Locatie AMC, Heart Centre, Amsterdam Cardiovascular Sciences, University of Amsterdam, Amsterdam, Netherlands; 2 General Practice Medicine, Amsterdam UMC - Locatie VUmc, Vrije Universiteit, Amsterdam Public Health Research Institute, Amsterdam, Netherlands; 3 Computer Science, Vrije Universiteit Amsterdam, Amsterdam, Netherlands; 4 Cell and Chemical Biology, Leiden University Medical Center, Leiden, Netherlands; 5 Biomedical Data Sciences, section Molecular Epidemiology, Leiden University Medical Centre, Leiden, Netherlands; 6 Epidemiology and Data Science, Amsterdam UMC, Amsterdam Public Health Research Institute, Amsterdam, Netherlands; 7 PHARMO Institute, Utrecht, Utrecht, Netherlands; 8 Department of General Practice, Amsterdam Public Health, Amsterdam UMC Locatie AMC, Amsterdam, Netherlands; 9 Netherlands Heart Institute, Utrecht, Netherlands

**Keywords:** ventricular fibrillation, heart arrest, electronic health records, diabetes mellitus, epidemiology

## Abstract

**Introduction:**

Early recognition of individuals with increased risk of sudden cardiac arrest (SCA) remains challenging. SCA research so far has used data from cardiologist care, but missed most SCA victims, since they were only in general practitioner (GP) care prior to SCA. Studying individuals with type 2 diabetes (T2D) in GP care may help solve this problem, as they have increased risk for SCA, and rich clinical datasets, since they regularly visit their GP for check-up measurements. This information can be further enriched with extensive genetic and metabolic information.

**Aim:**

To describe the study protocol of the REcognition of Sudden Cardiac arrest vUlnErability in Diabetes (RESCUED) project, which aims at identifying clinical, genetic and metabolic factors contributing to SCA risk in individuals with T2D, and to develop a prognostic model for the risk of SCA.

**Methods:**

The RESCUED project combines data from dedicated SCA and T2D cohorts, and GP data, from the same region in the Netherlands. Clinical data, genetic data (common and rare variant analysis) and metabolic data (metabolomics) will be analysed (using classical analysis techniques and machine learning methods) and combined into a prognostic model for risk of SCA.

**Conclusion:**

The RESCUED project is designed to increase our ability at early recognition of elevated SCA risk through an innovative strategy of focusing on GP data and a multidimensional methodology including clinical, genetic and metabolic analyses.

**Key questions**

**What is already known about this subject?**

- Early recognition of individuals with increased risk of sudden cardiac arrest (SCA) remains challenging. SCA research so far has used data from cardiologist care, but missed most SCA victims, since they were only in general practitioner (GP) care prior to SCA.

**What does this study add?**

- To achieve early recognition of most SCA victims, the REcognition of Sudden Cardiac arrest vUlnErability in Diabetes (RESCUED) project will address patients at elevated SCA risk in the primary care setting. We will focus on diabetes patients as they have increased risk for SCA, and also regularly visit their GP for check-up measurements. Moreover, we will enrich this information with genetic and metabolic information from two dedicated cohorts (SCA and diabetes) in the same study region, and analyses will include classical techniques and machine learning methods.

**How might this impact on clinical practice?**

- The RESCUED project is designed to discover new predictors of SCA and build a risk score, which will increase our ability for early recognition of individuals in the primary care setting with elevated SCA risk and facilitate the development of preventative strategies.

## Introduction

Sudden cardiac arrest (SCA) is a significant general health problem, causing 50% of cardiac deaths^1 2^ and around 20% of all natural deaths in Europe.^3^ SCA mostly occurs in an out-of-hospital setting and treatment often arrives too late. Only 6%–20% of SCA victims survive and survivors may suffer significant residual disability.^4^ Clearly, prevention is crucial. This requires timely recognition of individuals at increased risk of SCA and therefore insight into predictors. To date, research efforts to discover such predictors have focused on high-risk patients that are typically seen in cardiology practice,^5^ but these efforts have yielded only few predictors. Moreover, most SCA events occur in individuals whose risk was perceived to be low, both by themselves and by their general practitioners (GPs),^6 7^ and who have not received cardiologic investigation before SCA struck, and consequently lack cardiologic records.^8^ Clearly, the exploration of new research strategies is needed.

These considerations were the basis for the design of the REcognition of Sudden Cardiac arrest vUlnErability in Diabetes (RESCUED) project. This project aims to discover novel predictors of SCA in individuals with type 2 diabetes mellitus (T2DM) and has as key innovation that it is focused on individuals in Dutch GP practice. The focus on T2D stems from the high (and increasing) prevalence of T2D, the observation that SCA risk is twofold increased in T2D^9 10^—and already increased in individuals with glucose intolerance without overt T2D^9 11 12^—and the fact that people with T2D have rich GP datasets as they regularly visit the GP for structured T2D care. Moreover, realising that current methods of discovering SCA predictors—typically, logistic regression models based on clinical data—have limited power, state-of-the-art methods that use recent technical advancements will also be used: machine learning analysis of clinical data (including text mining), genetics and metabolomics.

**Figure 1:**
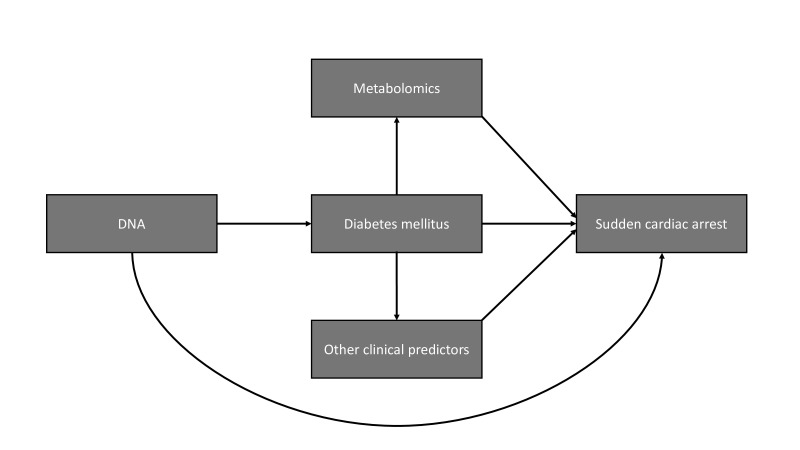
Risk factors contributing to sudden cardiac arrest occurrence studied within rescued.

The aim of this paper is to describe the rationale, outline and design of the RESCUED project. The RESCUED project has three main objectives: (1) to discover clinical predictors of SCA in T2D through systematic study of dedicated SCA and T2D cohorts and GP files, using regression analyses and machine learning methods; (2) to discover the (molecular) mechanisms that underlie SCA risk in T2D through DNA and metabolomics studies, as SCA has a genetic component,^13^ while metabolic factors may play a role in SCA development in T2D^14^ and (3) to recognise people with T2D at increased SCA risk early through the design of a risk score for SCA in T2D (figure 1).

## Methods

### Data collection

Study participants in RESCUED are drawn from three sources: the Amsterdam Resuscitation Studies (ARREST) registry, the Hoorn Diabetes Care System (DCS) and electronic GP files.

The ARREST registry is an ongoing prospective community-based registry of all out-of-hospital resuscitation events with involvement of Emergency Medical Services in the province North Holland of the Netherlands (urban and rural areas, population 2.4 million).^15^ All consecutive SCA patients in this study region have been included in this registry since 2007. ECGs are collected to ascertain a cardiac cause of the SCA, defined as ECG documentation of ventricular tachycardia/fibrillation (VT/VF). In addition, medical history, complete drug use in the year preceding SCA, and resuscitation variables are collected, along with blood samples for DNA and metabolomics studies. For RESCUED, only patients with ECGs-documented VT/VF and T2D are analysed.

**Figure 2:**
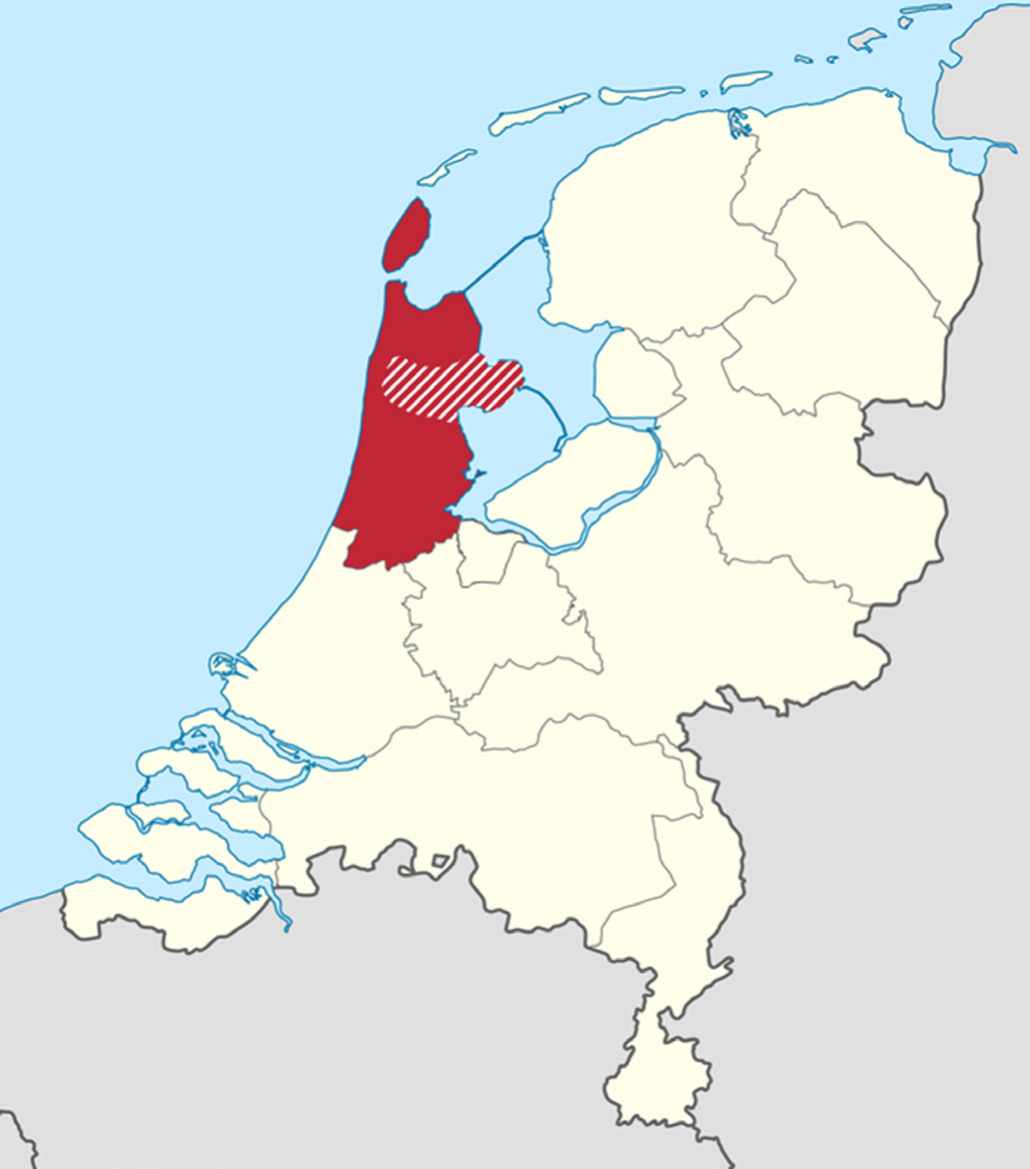
Study regions of included cohorts. The red area shows the province of North Holland (ARREST). The red striped area within the red area shows the region of West-Friesland (DCS). ARREST, Amsterdam Resuscitation Studie; DCS, diabetes care system.

The DCS cohort is an ongoing longitudinal cohort study which started in 1998. The DCS provides centralised and standardised diabetes care for people with T2D (n>15 000, biobank samples available for n=~5000) of collaborating GPs from the West-Friesland region of the Netherlands. Participants of the DCS cohort visit the DCS research centre annually for standardised check-up measurements as part of routine diabetes care.^16^ Comprehensive information about disease progression, microvascular and macrovascular complications and mortality is collected (eg, annual ECGs and longitudinal biochemical, anthropometric and medication data), along with a biobank that can be used for DNA and metabolomics analysis. This is a unique data resource, because the DCS region lies entirely within the ARREST region (figure 2); thus, all SCA victims of the DCS cohort are included in the ARREST registry. Of these participants, rich longitudinal data prior to the SCA event is collected in a standardised fashion and is available.

**Figure 3:**
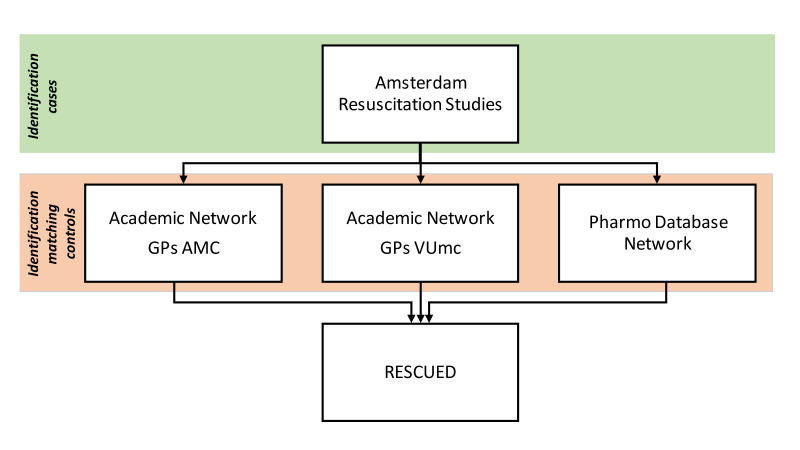
Data collection of medical history.

For each SCA victim in ARREST with T2D, of whom data from their GP can be retrieved, up to five anonymous sex and age matched non-SCA controls with T2D will be drawn from the same GP practice. Since the early 1990s, GP files in the Netherlands have progressively become fully electronic. These electronic files contain a complete overview of a patient’s medical history (including cardiologist’s and hospital discharge letters if present), because in the Dutch healthcare system the GP is the gatekeeper for specialist medical care. To obtain these electronic GP files, the RESCUED consortium will use three existing networks; (1) Academic Network of GPs of Amsterdam UMC location VUmc (ANH) (2) Academic Network of GPs of Amsterdam UMC location AMC (AHA) and (3) PHARMO Database Network (figure 3). The ANH and AHA networks are networks of GP practices that have been established by the Departments of GP Medicine of both academic hospitals of Amsterdam. Collaborating GPs contribute their patient data to the network database for research purposes. The PHARMO Database Network is a population-based network of electronic healthcare databases from different primary and secondary healthcare settings, including GP practices. To ensure the privacy of the data, the collection, processing, linkage and anonymisation of the data is performed by the Foundation for Information Provision for Care and Research (STIZON). STIZON is an independent, ISO/IEC 27001 certified foundation, which acts as a Trusted Third Party. The RESCUED consortium plans to link GP practices in the study region who are not yet linked to any of these three networks to the PHARMO Database Network, ensuring uniform data processing.

### Clinical data

From the GP files, information from 2 to 5 years before the SCA will be extracted. This information includes International Classification of Primary Care (ICPC) and Anatomical Therapeutic Chemical (ATC) codes for medications, episodes, consults, lifestyle, treatment, laboratory results, anthropometric measures, referrals to specialists and free-text consultation notes.

### DNA data

DNA samples collected in the ARREST and DCS cohorts will be used.^15 16^ In ARREST, DNA of SCA victims is isolated from residual material left over from blood collection used for routine patient care.^15^ In the biobank subsample of DCS, DNA from people with T2D has been isolated from blood samples taken during visits to DCS for routine diabetes care. Genotyping for genome-wide association studies (GWAS) is done with the Illumina Human CoreExome array (DCS) or the Illumina Global Screening Array (DCS, ARREST).

### Metabolomics data

The plasma samples needed for metabolomics analysis of SCA cases are obtained from the same blood samples that are used for DNA isolation and stored at −80°C. For the controls, the plasma samples were previously collected in DCS and are stored at −80°C. All samples will be analysed on the 1H-NMR platform (Nightingalehealth.com) that allows comprehensive research into the interplay of health, lifestyle factors and genetics for future SCA risk and has been used in many epidemiological studies.^17^ These methods provide simultaneous quantification of routine lipids, lipoprotein subclass profiling with lipid concentrations within 14 subclasses, fatty acid composition and various low-molecular metabolites, including amino acids, ketone bodies, and gluconeogenesis-related metabolites, in molar concentration units. Several of the metabolic biomarkers have already been validated with other techniques.^18^

### Data analysis

#### To discover clinical predictors of SCA

As first step, we will compare clinical information of all SCA cases with T2D to age and sex-matched non-SCA controls with T2D using multiple univariable and multivariable logistic regression analyses with ORs and 95% CIs. Candidate variables will be formulated based on literature and medical expert opinion. If necessary, correlation and/or univariable logistic regression analyses will be used for pruning of candidates for the multivariable models. One of the options that we will explore is to use a backward selection procedure with bootstrapping to find the most informative predictors. In addition, we will perform machine-learning based analysis using both coded data (ICPC/ATC) and free text from the GP files (eg, consultation notes). As a process model to successfully apply these machine learning techniques, we will use cross-industry standard process for data mining (CRISP-DM).^19^ In this process, we will exploit a variety of state-of-the-art machine learning algorithms, including methods that allow us to take advantage of temporal patterns observed in the data (eg, a certain diagnosis preceding another diagnosis). On top, we will use a variety of text mining approaches to extract useful variables (or ‘features’ in machine learning terms) from the uncoded consultation notes. These will include topic modelling such as Latent Dirichlet Allocation^20^ and ‘standard’ bag of words based approaches and will be fed into the aforementioned machine learning algorithms as extra features. We have previously shown that such methods enable us to extract useful predictors from the coded data as well as text (uncoded) consultation notes, while improving predictive performance over more traditional statistical methods.^21 22^ We will exploit decision trees,^23^ random forests,^24^ gradient boosting^25^ and neural networks,^26^ providing a set of methods that range from more insightful models to more black box models. For the latter, we will use dedicated techniques such as Shapley additive explanations (SHAP)^27^ and local interpretable model-agnostic explanations (LIME)^28^ to gain insights into the models. The data will be split into a training and test set, which enables us to internally validate the predictive model in other patients. The suitability of the different machine learning techniques that we apply will be assessed and tested on the test set. Feedback by medical experts will be given, and using this feedback a new series of models will be made. The performance of the models will be validated by assessing measures of calibration and discrimination and we will compare the outcome to the outcome of the classical multivariable logistic regression analyses. In the end, models with clear predictors for SCA will result.

#### To discover genetic predictors of SCA

Recent studies have shown the association of common genetic variants with ECG parameters and SCA risk.^29–32^ The aims of the various GWAS approaches in this project are to replicate these associations in SCA victims, and also to identify novel associations, potentially unique to T2D. To reach these aims, we will compare T2D SCA victims with (1) T2D non-SCA controls, (2) SCA victims without T2D and (3) the general population. Furthermore, we will assess the additional value of a Genetic Risk Score (GRS) combining the genetic information of multiple risk loci, in the final risk model. As common variants can be typed at relatively low costs (~€30/patient), a GRS is a potential additional risk factor that could be implemented in clinical practice. In addition to the GWAS, we will evaluate the presence of rare genetic variants using next-generation sequencing methods (eg, whole exome sequencing or whole genome sequencing) in SCA cases in whom a significant genetic contribution to SCA is likely, for example, because of young age and/or absence of T2D complications associated with increased SCA risk, such as acute myocardial infarction. If these analyses should reveal unexpected actionable findings (disease-causing mutations), the GP will be contacted with the request to inform the patient, and the advice to refer the patient for genetic counselling.

#### To discover metabolomics predictors of SCA

Metabolomics profiles will be compared between SCA cases and non-SCA controls to study whether changes in metabolites are associated with SCA occurrence using univariable and multivariable regression models adjusted for potential confounders. In addition, we aim to identify the metabolomics changes in persons with T2D that precede the occurrence of SCA. In DCS we used longitudinal sampling which allows us to study temporal changes in metabolite profiles prior to the SCA event.

#### To develop a prediction model for SCA

Using the clinical, genetics and metabolomics data, a prognostic model for risk of SCA will be developed. A backward selection strategy will be used to select the most parsimonious clinically relevant model. Interactions between risk factors will be investigated. Bootstrapping will be performed to correct for optimism, as models usually perform best in their development data sets. The performance of the models will be evaluated by studying the calibration of the models by comparing the predicted number of events with the observed number of events. Moreover, the discrimination of the models will be tested (ability to distinguish between patients who did or did not have an SCA). Performance of different prediction models (with or without interactions) will be compared with reclassification statistics, which takes diversity into account. On top, machine learning models will be developed following a similar approach as described before, but with extended data in the form of the genetic and metabolomics data. Data of SCA victims collected in the ESCAPE-NET project^33^ will be used to externally validate the risk score.

### Sample size calculations

#### Clinical predictors

The total number of patients that can be used to study clinical predictors depends on the availability of the electronic patient files. SCA cases will be matched to, on average, three age-matched and sex-matched non-SCA controls. We aim to include 1000 cases, because this number will give us 90% power at 5% significance level to detect an OR of 1.34. In case we can only include 500 cases, the significant OR we can detect at 90% power and 5% significance level is 1.51.

#### Genetic predictors

To conduct GWAS, we expect to study 800 SCA cases with T2D and an equal number of age and sex matched non-SCA controls with T2D. With these numbers, we will have 80% power to detect variants with an OR of ≥4.3, ≥2.2, ≥1.8 and ≥1.5 with a minor allele frequency of 0.01, 0.05, 0.1 and 0.5, respectively, at a genome wide significance level α=5×10^−8^, using an additive model.^34^ Of note, multiple publications by our group indicate that in situations with deeply phenotyped patients such as our cohorts, GWAS with relatively limited numbers is possible and can yield very interesting novel loci.^29 35^

To conduct whole exome sequencing or whole genome sequencing analysis, the power calculation to detect rare pathogenic variants underlying SCA in patients with T2D is independent of sample number as this analysis is performed on the individual patient level. The expected power for burden testing is low. However, considering the potential presence of large-effect alleles with a founder effect in the Netherlands^36^ and the presence of matched controls within our department, it is worthwhile to pursue this line of investigation.

#### Metabolomic predictors

With 400 expected SCA cases with T2D and 1000 non-SCA T2D controls, we have 80% power to detect a difference in standardised metabolite levels of around 0.245 at alpha=0.001. For similar numbers of cases of T2D, we can find >50 metabolite associations with p<0.0001 (in age, sex, fasting glucose adjusted models).

#### Prediction modelling

For model development, 10–20 events per predictor are suggested.^37–40^ The 400 expected SCA events in the data from ARREST (with all available info), will provide sufficient power to include 20-40 candidate predictors in the model. However, there are no operational sample size calculations available for the combination of machine learning techniques we plan to exploit. Nevertheless, an earlier publication with similar sample size yielded adequate power.^21^

## Results

**Figure 4:**
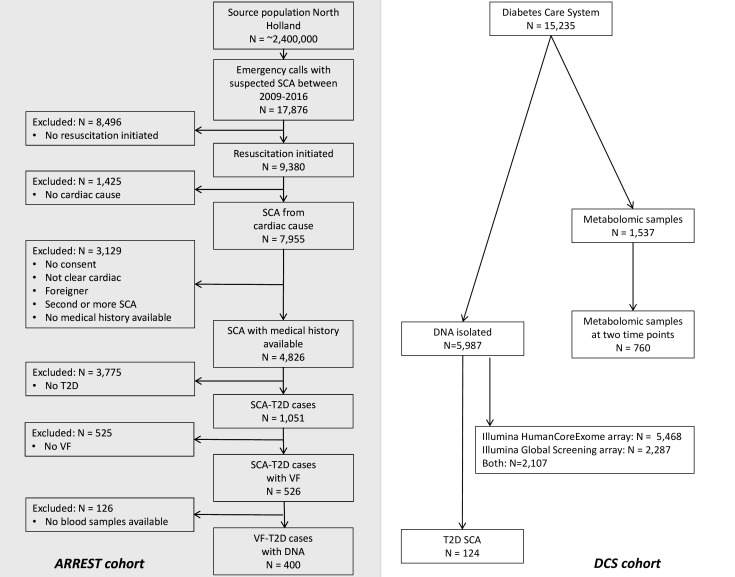
Flow chart of patient inclusion from arrest and DCS cohorts. DCS, Diabetes Care System; SCA, sudden cardiac arrest; T2D, type 2 diabetes; VF, ventricular fibrillation.

Already available data from the ARREST and DCS cohorts are shown in the flow chart of figure 4. Nevertheless, the numbers indicated will increase as inclusion is ongoing. The ARREST registry has collected 1051 SCA cases with T2D in the years 2009–2016. Of these cases, n=526 had documented VF and blood samples for genetic analysis are available in n=400. Collection of samples to be used for metabolomics analysis started in October 2018, and n=350 samples have been collected until September 2020. The DCS cohort has n=15 235 people with T2D of which 5987 have genetic samples and 1537 have metabolomics samples. Among those with genetic samples available, n=124 had suffered SCA. In those with metabolomics samples, n=760 have samples at two time points.

The ANH database contains data from 50 GP practices. The AHA database contains data from 46 GP practices. Approximately 100 T2D SCA patients from ARREST are included in the ANH and AHA databases. The PHARMO Database Network contains data from 69 GP practices in the study region, and data from 786 GP practices nationwide, with more to follow.

## Discussion

The recognition of people at increased risk of SCA remains difficult, as SCA is an extremely complex phenomenon that may result from a great variety of causes from different dimensions (e.g. comorbidities, genetic susceptibility, medication use, intercurrent factors) and different triggers. Each individual has a unique set of interacting causes that may be relatively innocuous in isolation, but that may cause SCA when combined. Early recognition requires a clear understanding of the predictive factors of SCA on an individual’s level. Given the large number of possible relevant factors, this requires the collection of large and comprehensive sets of data from the period that leads up to the SCA event. Ideally, this should include both clinical data and biosamples that allow for discovery of relevant molecular factors, for example, genetic and metabolic factors. However, due to the unpredictable occurrence of SCA, it is extremely difficult to gather such information. Recognising these requirements and difficulties to fulfil them, we created an infrastructure to collect clinical data from GP care for subsequent analysis with machine learning methods, and biosamples for DNA and metabolic analysis. Crucially, this infrastructure can also be used in future studies to discover SCA predictors for individuals from the general population in GP care without diabetes. This will allow us to discover predictors of SCA in the general population, and will increase our ability to identify individuals from the general population with an increased risk of SCA.

We recognise the following limitations. First, GP practices use different electronic patient data systems, which might lead to different methods of data entry. However, by using GP-matched controls (controls from the same GP practice as the cases), we expect to mitigate this problem. Second, observational cohorts are used, which have inherent limitations for inferring causality. Additionally, the blood plasma samples for our metabolomics analysis are samples taken after the SCA event and have a delay in processing (between blood collection and freezing). This might result in a disturbed metabolite profile. However, previous research with this platform has observed that most metabolites (especially lipid related) are minimally affected by the prestorage and poststorage conditions tested.^41^ Although we have data of many patients at our disposition, prediction of a complex and multicausal phenomenon like SCA might demand even bigger numbers.

## Conclusion

The RESCUED project will use an innovative methodology to discover predictors and molecular mechanisms of SCA that may be used for better early recognition of SCA risk in individuals with T2D. The RESCUED project is specifically designed to extract maximal information from GP files, a data source with great potential, which has remained largely untapped in SCA research.
